# Redeployment experiences of healthcare workers in the UK during COVID-19: a cross-sectional analysis from the nationwide UK-REACH study^*^

**DOI:** 10.1177/20542704241290721

**Published:** 2024-10-30

**Authors:** Zainab Zuzer Lal, Christopher A. Martin, Mayuri Gogoi, Irtiza Qureshi, Luke Bryant, Padmasayee Papineni, Susie Lagrata, Laura B Nellums, Amani Al-Oraibi, Jonathon Chaloner, Katherine Woolf, Manish Pareek

**Affiliations:** 1 Department of Respiratory Sciences, 4488 University of Leicester, Leicester, UK; 2 Development Centre for Population Health, 4488 University of Leicester, Leicester, UK; 3 Department of Infection and HIV Medicine, University Hospitals of Leicester NHS Trust, Leicester, UK; 4 National Institute of Health Research (NIHR), Leicester Biomedical Research Centre (BRC), Leicester, UK; 5 Centre for Public Health and Epidemiology, 6123 University of Nottingham, Nottingham, UK; 6 Ealing Hospital, London North West University Healthcare NHS Trust, Southall, UK; 7 8964 University College London Hospitals NHS Foundation Trust, London, UK; 8 College of Population Health, University of New Mexico, Albuquerque, NM, USA; 9 University College London Medical School, London, UK; 10 National Institute of Health Research (NIHR), Applied Health Collaboration (ARC) East Midlands, Nottingham, UK

**Keywords:** occupational and environmental medicine, public health, statistics and research methods, respiratory medicine‌

## Abstract

**Objectives:**

To assess how ethnicity, migration status and occupation are associated with healthcare workers (HCW) redeployment experiences during COVID-19 in a nationwide ethnically diverse sample.

**Design:**

A cross-sectional analysis using data from the nationwide United Kingdom Research Study into Ethnicity And COVID-19 outcomes in Healthcare workers (UK-REACH) cohort study.

**Setting:**

Healthcare settings.

**Participants:**

Healthcare workers (HCW).

**Main Outcome Measures:**

Outcome measures included redeployment, provision of training and supervision during redeployment, change in patient contact and interaction with COVID-19 patients.

**Methods:**

We used logistic regression to examine associations of ethnicity, migration status, and occupation with redeployment experiences of HCWs.

**Results:**

Of the 10,889 HCWs included, 20.4% reported being redeployed during the first UK national lockdown in March 2020. Those in nursing roles (Odds Ratio (OR) 1.22, 95% Confidence Interval (CI) 1.04–1.42, *p* = 0.009) (compared to medical roles) had higher likelihood of being redeployed as did migrants compared to those born in the UK (OR 1.26, 95% CI 1.06–1.49, *p* = 0.01) (in a subcohort of HCWs on the agenda for change (AfC) pay scales). Asian HCWs were less likely to report receiving training (OR 0.66, 95% CI 0.50–0.88, *p* = 0.005) and Black HCWs (OR 2.02, 95% CI 1.14–3.57, *p* = 0.02) were more likely to report receiving supervision, compared to White colleagues. Finally, redeployed Black (OR 1.33, 95% CI 1.07–1.66, *p* = 0.009) and Asian HCWs (OR 1.30, 95% CI 1.14–1.48, *p* < 0.001) were more likely to report face-to-face interaction with COVID-19 patients than White HCWs.

**Conclusions:**

Our findings highlight disparities in HCWs’ redeployment experiences by ethnicity, migration, and job role which are potentially related to structural inequalities in healthcare.

The COVID-19 pandemic has caused over 6 million deaths worldwide as of August 2023.^1^ People from ethnic minority backgrounds have had a significantly higher risk of contracting COVID-19 and dying from it.^2^ In the UK, COVID-19 mortality rates have been higher in Bangladeshi, Pakistani and Black Caribbean communities compared to the White British group.^3^ Front-line healthcare workers (HCWs) have also faced a disproportionate risk of COVID-19 compared to the general population, with a previous study finding a threefold higher risk of SARS-CoV-2 infection which may have been exacerbated by the inadequate availability of personal protective equipment (PPE).^4–7^ The intersection of heightened risk and increased rates of infection in ethnic minority groups may be mediated by occupational and sociodemographic factors, which, in turn, are manifestations of structural discrimination.^8,9^ These factors include inhabiting densely populated areas, living in multigenerational households, and occupying housing with poorer ventilation.^10,11^ Therefore, within ethnic minority HCWs, the convergence of ethnicity and occupation puts them at higher risk of COVID-19 infection, morbidity and mortality.^8,12^ The impact of the pandemic on healthcare systems has also been profound, with acute staffing shortages, limited number of beds, growing waitlists and increased caseloads.^13^ In response to these escalating demands, various strategies were implemented in April 2020, including staff mobilisation, redeployment to areas outside their professional training, alteration of work schedules, and risk assessments to assess individual risk factors for COVID-19 and concerns of employees.^14,15^ Redeployment, specifically, was employed for reassigning HCWs to alternative units or speciality areas.^16^ Although redeployment played a critical role in effectively managing the crisis, recent research suggests it significantly affected staff well-being, with over 95% HCWs reporting stress and anxiety after being redeployed.^17^ Previous studies have highlighted ethnic inequalities in redeployment, with HCWs from ethnic minority backgrounds being more likely to be redeployed to COVID-19 areas than their White counterparts.^17,18^ However, these studies were conducted in small cohorts with a low proportion of participants from ethnic minority groups and did not examine outcomes relating to experiences of redeployment such as the degree of patient contact in the redeployed role or the receipt of training/supervision.^17,18^

Therefore, to address this knowledge gap, we conducted an analysis using data from a nationwide cohort study of UK HCWs, the UK-REACH Study (UK Research study into Ethnicity And COVID-19 outcomes in Healthcare workers). Our aims were to determine whether ethnicity, migration status or occupational role affected the likelihood of redeployment. Amongst those redeployed, we aimed to examine the receipt of training and supervision before/during redeployment, the level of direct patient contact in the redeployed role in comparison to the usual role, and whether the role involved COVID-19 contact.

## Methods

UK-REACH, a nationwide cohort study comprises multiple work packages to understand the impact of COVID-19 on HCWs from diverse ethnic backgrounds. Here, we used data from the baseline questionnaire of the study.

### Recruitment

We included HCWs (including ancillary workers in healthcare settings) aged 16 and above. The first stage of recruitment involved healthcare regulators (see Supplementary Information for a list of participating regulators) sending email invitations to their registrants. We directed interested HCWs to the UK-REACH website (https://www.uk-reach.org) where they could access the participant information sheet and provide informed consent. We then asked participants to complete the online questionnaire. We supplemented this sample by direct recruitment from participating NHS Trusts (see study protocol and cohort profile for further details^19,20^).

We administered the baseline questionnaire between 4th December 2020 and 8^th^ March 2021.

### Defining the analysed cohort

**Figure 1.**
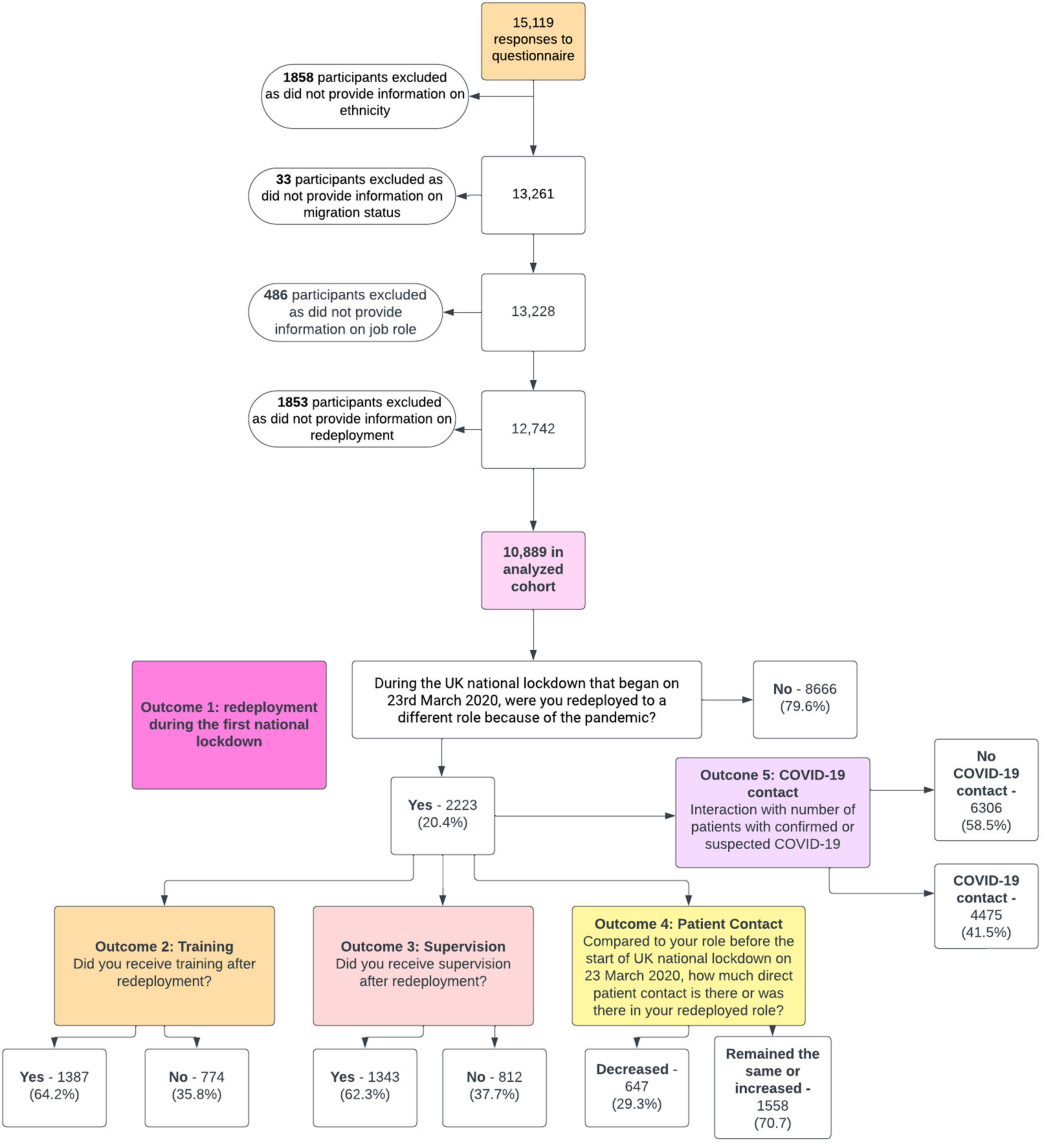
Formation of the analysed sample and derivation of outcome measures.

Formation of the analysed sample is shown in Figure 1.

We excluded participants who did not provide information on ethnicity, migration status, occupation, and redeployment. We asked participants about redeployment experiences during the first UK national lockdown and therefore excluded anyone who indicated they were not working during this period. To ensure our measures of COVID-19 work patterns reflected experiences at the time of staff deployment, we used answers to questions about occupational circumstances in the weeks following the UK national lockdown (referred to as ‘the first UK national lockdown on 23 March 2020’, throughout the questionnaire).

### Outcome measures

We derived 5 binary outcome measures from questionnaire items (Figure 1). Outcome 1 is derived from the question ‘During the UK national lockdown that began on 23rd March 2020, were you redeployed to a different role because of the pandemic?’. Outcomes 2 and 3 are derived from the questions ‘Did you receive training during redeployment?’ and ‘Did you receive supervision during redeployment?’. Outcome 4 is derived from the question ‘Compared to your role before the start of UK national lockdown on 23 March 2020, how much direct patient contact is there or was there in your redeployed role?’. Outcome 5 is not derived from a question that directly relates to redeployment, but rather a questionnaire item asking HCWs to declare the number of face-to-face interactions with COVID-19 patients in a typical week during the first month after the start of the lockdown (see Figure 1 for further details). Both, redeployment and the COVID-19 patient contact items ask about the same period and it is assumed therefore that HCWs who were redeployed provided information on COVID-19 patient contact in their redeployed roles.

- Outcome 1: redeployment (redeployed vs not redeployed);
- Outcome 2: received training during redeployment (received vs did not receive);
- Outcome 3: received supervision during redeployment (received vs did not receive);
- Outcome 4: change in patient contact during redeployment (stayed the same or decreased vs increased);
- Outcome 5: interaction with number of patients with confirmed or suspected COVID-19 (no COVID-19 patient contact vs COVID-19 patient contact).

For analysis of outcomes 2, 3, 4 and 5, we only included those who answered they were redeployed.

### Exposures

Our exposures of interest were self-reported ethnicity, migration status and occupation.

- **Ethnicity:** categorised into five broad ethnic groups, as suggested by the UK Office for National Statistics (White, Asian, Black, Mixed and Other),^21^ to increase statistical power.
- **Migration status**: categorised as born in the UK or outside the UK, following the Migration Observatory's definition of migrants based on their country of birth.^22^
- **Occupation**: categorised as doctors, nursing staff (including midwives and nursing associates), allied health professionals, pharmacy staff, healthcare scientists, ambulance staff, dental, optical, administrative, estates, facilities or other wider healthcare roles and others (including medical associates)

### Covariates

Based on existing literature and expert opinion, potential confounders of the relationship between our exposure and outcome measures were hypothesised to be:

- **Demographic characteristics** (age categorised into 16–30-year-olds, 31- 45, 46–60 and 61 and above; sex categorised into male and female),
- **Occupational factors** (job sector – i.e. worked for the NHS in any capacity, or worked outside the NHS)
- **Deprivation** – measured by the index of multiple deprivation based on individual HCWs residential postcode area (IMD, the official measure of relative deprivation for small areas or neighbourhoods in England, expressed as quintiles)^23^
- **Self-reported long-term health conditions** (LTCs) categorised as those requiring shielding – including organ transplant, diabetes, asthma, heart disease, kidney disease, liver disease, cancer, and immunosuppression (based on the COVID-19 guidance for people who were considered at higher risk),^24^ other conditions (including hypertension, obesity, stroke, conditions affecting the brain or nervous system and mental health conditions), and no conditions.

### Subgroup analyses

To control for the effect of more granular occupational variables and occupational seniority we conducted analyses on two subgroups; doctors and those on the NHS agenda for change (AfC) pay scales. The NHS AfC pay bands comprise bands 1–9 (with salary increasing as band level rises) for HCWs other than doctors, dentists and very senior managers. This scale was used as a proxy measure for occupational seniority. We repeated analyses of outcomes 1, 2 and 3 within these subgroups adjusting for the grade or stage of training for doctors and the AfC pay band for those on these pay scales.

### Statistical analysis

All variables were categorical were summarised as frequency and percentage. The derivation of all variables used in the analysis is highlighted in Supplementary Table S1.

We used univariable and multivariable logistic regression models to explore relationships between the exposure and outcome variables. The results are presented in terms of odds ratios (ORs) and adjusted odds ratios (aORs), 95% confidence intervals (CIs), and *p*-values. In this analysis, we have focused on presenting effect sizes and their 95% confidence intervals to provide a better understanding of the strength of the associations instead of dichotomising *p*-values by arbitrary cut-offs.^25,26^

Adjusted models included ethnicity, occupation and migration status, along with confounders (age, sex, LTCs and deprivation). In subgroup analyses of specific occupational groups, we added AfC pay band (for those in non-medical roles) and grade (for doctors). For analysis of outcomes 2 and 3, we did not include the underlying comorbidities variable since we believed they would not impact whether HCWs received training and supervision during redeployment.

All analyses were conducted using Stata V.17.

### Missing data

We presented the frequency and proportion of missing data for each variable used in the analysis. We used multiple imputation by chained equations to impute missing data in the logistic regression models. In the imputation models, we included all variables used in the analysis of outcome 1 (including the outcome measure), except the one being imputed. We applied Rubin's Rules to combine parameter estimates and standard errors from 10 imputations into a single set of results.^27^ We used a random number seed to ensure reproducible results. In subgroup analyses including grade or pay band, we excluded those who did not provide this information.

### Ethics

The study was approved by the Health Research Authority (Brighton and Sussex Research Ethics Committee; ethics reference: 20/HRA/4718). All participants gave written informed consent.

### Involvement and engagement

A Professional Expert Panel of HCWs from a diverse range of ethnic backgrounds, healthcare roles, and genders, both locally and nationally, worked closely to help develop the research question, analysis plan and manuscript.

### Role of the funding source

Funders had no role in the study design, data collection, analysis, interpretation, or writing of this report.

## Results

### Recruitment and formation of analysis sample

Figure 1 shows the formation of the analysed sample. In total, 15,119 HCWs responded to the questionnaire. After excluding 4230 participants due to missing data on ethnicity, migration status, job role, and redeployment (as detailed in Figure 1) – we arrived at our final analysis sample of 10,889 HCWs.

### Description of the analysed cohort

Table 1 summarises the analysed cohort in terms of demographic, household, and occupational factors together with the amount of missing data for each variable. The majority of those included were women (75.2%) and 30.0% were from ethnic minority groups (19.0% Asian, 4.2% Black, 4.1% Mixed, 2.0% Other). Approximately 25% were doctors, 23% worked in nursing and midwifery roles, and 30% in allied health professional roles.

**Table 1.** Description of the analysed cohort.

Variable	Description *N* = 10,889
**Demographic and household factors**	
** Ethnicity**	
White	7683 (70.6)
Black	460 (4.2)
Asian	2076 (19)
Mixed	448 (4.1)
Other	222 (2)
** Age**	
<30 years	1596 (14.7)
31–45 years	3987 (36.1)
46–60 years	4319 (39.7)
61 years and above	933 (9)
Missing	54 (0.5)
** Sex**	
Male	2676 (24.6)
Female	8187 (75.2)
Missing	26 (0.2)
** Migration status**	
Born in the UK	8000 (73.5)
Born overseas	2889 (26.5)
** Index of multiple deprivation (quintiles)**	
1 (most deprived)	951 (9.9)
2	1624 (16.9)
3	1971 (20.5)
4	2339 (24.3)
5 (least deprived)	2740 (28.5)
Missing	1264 (11.6)
**Underlying health conditions**	
No health conditions	4573 (42)
Conditions that required shielding during COVID-19^a^	2107 (19.4)
Other conditions	3232 (29.7)
Missing	977 (9.0)
**Occupational factors**	
** Occupation**	
Doctors	2688 (24.7)
Nursing	2485 (22.8)
Allied health professionals	3307 (30.4)
Pharmacy	233 (2.1)
Healthcare scientist	532 (4.9)
Ambulance	442 (4.1)
Dental	435 (4)
Optical	117 (1.1)
Administrative	622 (5.7)
Estates/facilities	103 (1)
Other	320 (3)
** Job sector**	
Worked for the NHS in any capacity	9056 (83.2)
Worked outside the NHS	1387 (12.7)
Missing	446 (4.1)
**Redeployment experiences**	
Redeployment	
Redeployed	2223 (20.4)
Not redeployed	8666 (79.6)
** Training redeployed staff**	
Received training	1387 (64.2)
Did not receive training	774 (35.8)
** Supervising redeployed staff**	
Received supervision	1343 (62.3)
Did not receive supervision	812 (37.7)
** Patient contact during redeployment**	
Decreased	647 (29.3)
Remained the same or increased	1558 (70.7)
**COVID-19 contact**	
No COVID-19 contact	6306 (58.5)
COVID-19 contact	4475 (41.5)

This table provides a description of the 10,889 HCWs who worked during the first UK national lockdown starting 23^rd^ March 2020. These HCWs provided information on their ethnicity, migration status, job role and answered questions about redeployment. All data in the right-hand column are *n* (%).

HCWs: healthcare workers; NHS: National Health Service.

^a^ Include organ transplant, diabetes, asthma, heart disease, kidney disease, liver disease, cancer and immunosuppression (based on the COVID-19 guidance^18^ for people who were considered at higher risk).

### Univariable analysis of predictor variables

Univariable analysis of redeployment is shown in Supplementary Table S3, while Supplementary Table S4 presents the analysis in a sub-cohort of doctors and HCWs on the AfC pay scale to control for seniority. Overall, 2223 (20.4%) of the 10,889 HCWs who worked during the lockdown reported being redeployed. HCWs from Mixed ethnic groups, as compared to those from White groups had higher odds of reporting redeployment (OR 1.28, 95% CI 1.03–1.60, *p* = 0.03). HCWs working in pharmacy (0.41, 0.27–0.63, *p* < 0.001), healthcare scientist (0.43, 0.32–0.57, *p* < 0.001), ambulance (0.5, 0.37–0.67, *p* < 0.001), dental (0.75, 0.57–0.97, *p* = 0.03), optical (0.53, 0.30–0.91, *p* = 0.02) and administrative (0.46, 0.30–0.70, *p* < 0.001) roles were less likely to report redeployment compared to doctors.

### Multivariable analysis

#### Redeployment

**Figure 2.**
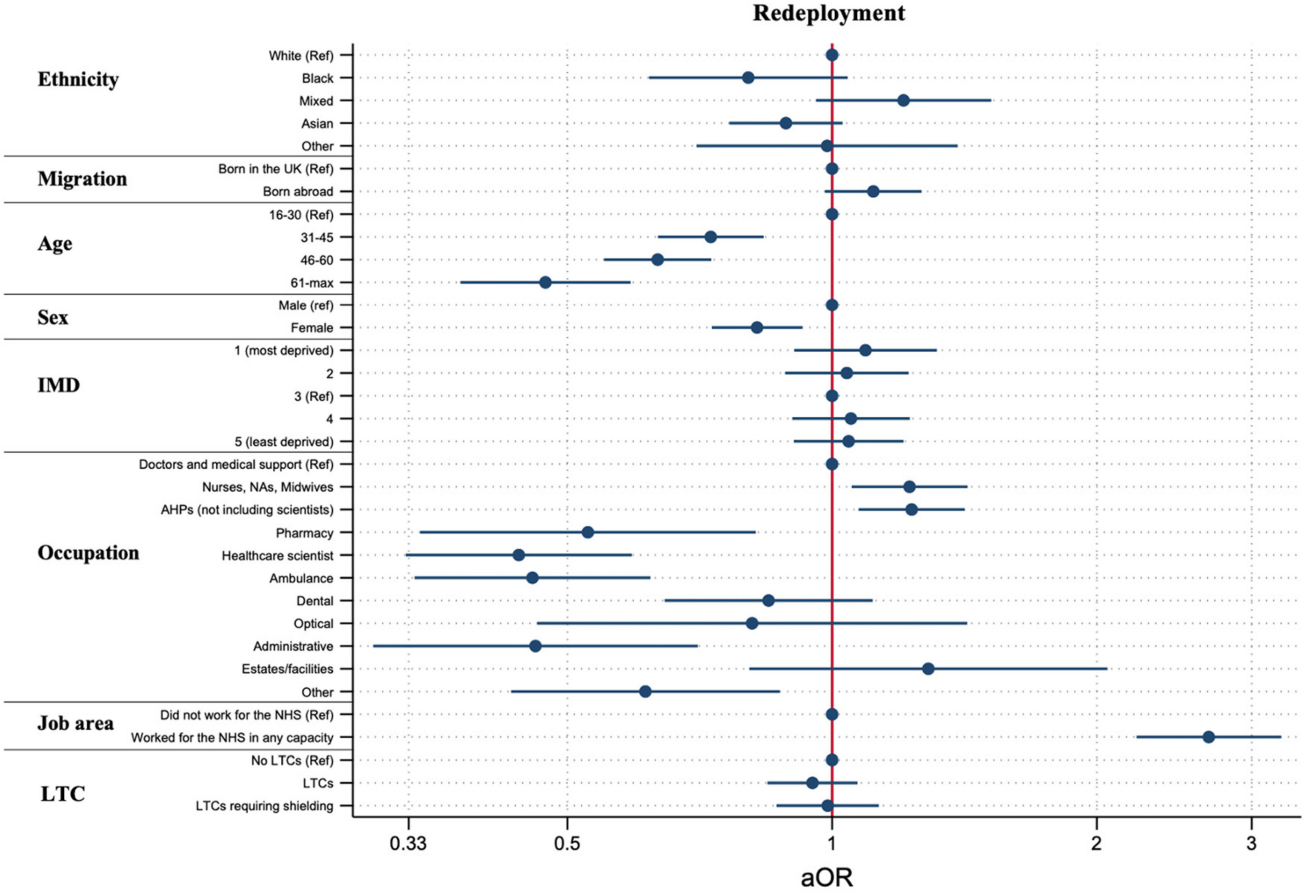
The relationship between ethnicity, migration status and occupation with redeployment after adjustment for demographic and health covariates.

Ethnic differences seen in the univariable model attenuated after adjustment for covariates (Figure 2 and Supplementary Table S4). Compared to doctors, those working in nursing and midwifery (1.22, 1.04–1.42, *p* = 0.009), and allied health professional roles (1.23, 1.07–1.41, *p* = 0.003) were more likely to report being redeployed.

#### Training and supervision in redeployed roles

**Figure 3.**
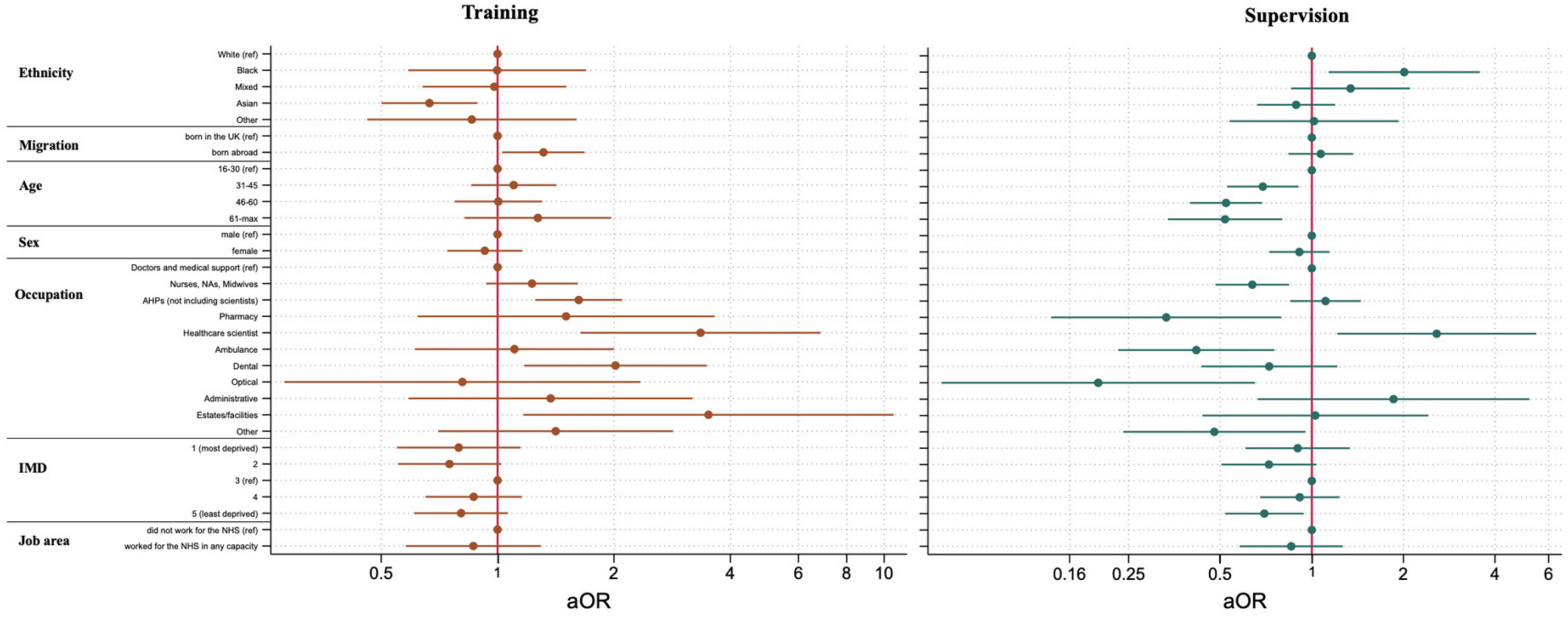
The relationship between ethnicity, migration status and occupation with training and supervision after adjustment for demographic and health covariates.

Of the 2223 HCWs who reported being redeployed, 62 did not respond to follow-up questions about training and 68 did not respond to questions about supervision. Among the redeployed HCWs, 64.2% (*n* = 1387) reported receiving training while 62.3% (*n* = 1343) reported receiving supervision. Asian HCWs were less likely to report training during redeployment compared to White HCWs (0.66, 0.50–0.88, *p* = 0.005). HCWs born outside the UK were more likely to report receiving training than those born in the UK (1.30, 1.01–1.66, *p* = 0.04) (Figure 3 and Supplementary Table S4).

Black HCWs were significantly more likely to report being supervised in their redeployed role (2.02, 1.14–3.57, *p* = 0.02) compared to their White counterparts. Additionally, those living in the least deprived areas were less likely to report supervision than those from most deprived areas (IMD quintile 5: 0.71, 0.54–0.95, *p* = 0.02 vs quintile 1) (Figure 3 and Supplementary Table S4).

#### Patient contact during redeployment

**Figure 4.**
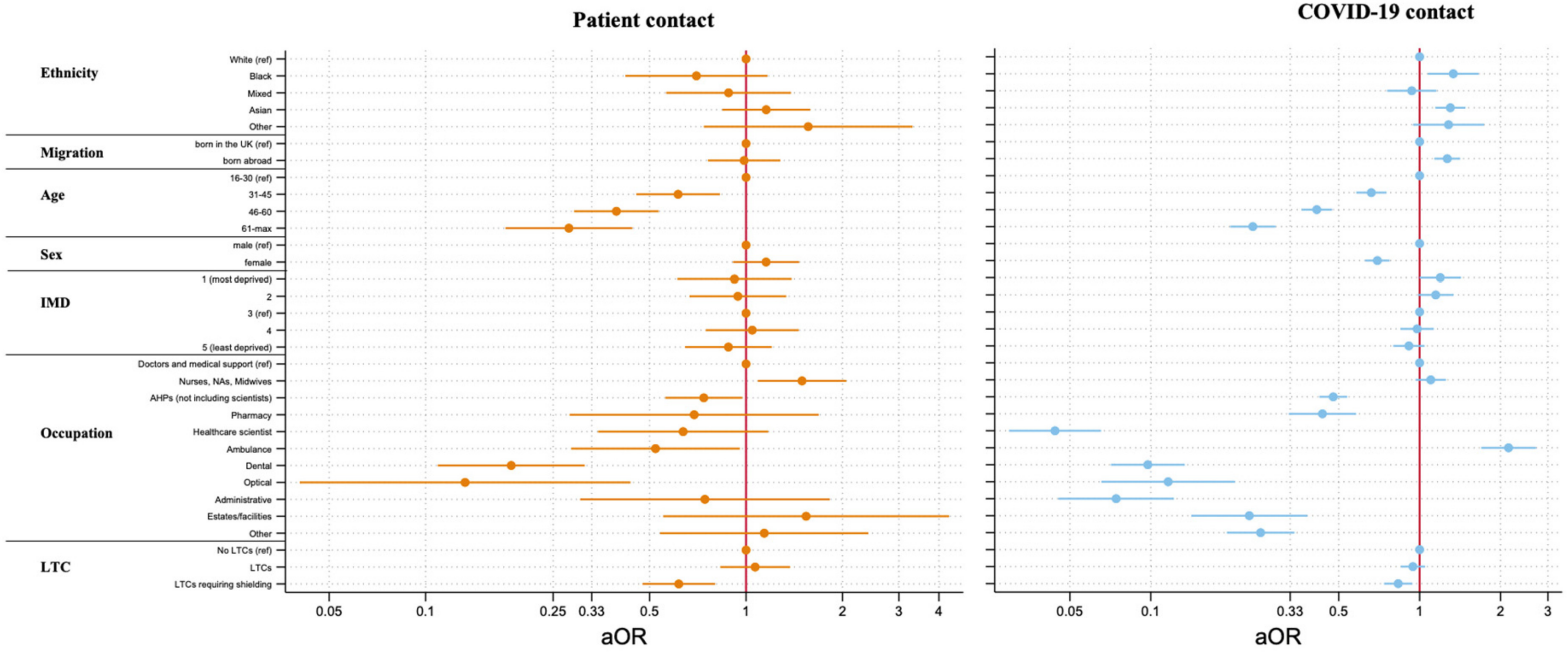
The relationship between ethnicity, migration status and occupation with patient contact and COVID-19 contact after adjustment for demographic and health covariates.

Nursing and midwifery roles, and allied health professional roles, in comparison to doctors, reported increased patient contact during redeployment (1.52, 1.1–2.08, *p* = 0.01), whereas most other HCW groups reported decreased patient contact in their redeployed roles. HCWs with LTCs that required them to shield during the pandemic had less patient contact during redeployment than those without such conditions (0.62, 0.48–0.79, *p* < 0.001) (Figure 4 and Supplementary Table S4).

Figures 2, 3 and 4 show adjusted ORs and 95% CIs, derived from multivariable logistic regression models, for the association of socio-demographic and occupational variables with 5 outcome measures relating to redeployment and redeployment experiences. ORs are adjusted for age, sex, ethnicity, occupation, migration, deprivation, and underlying long-term health conditions. All variables only include HCWs who were working during the UK national lockdown that began on 23^rd^ March 2020. The analysed sample included all HCWs who provided information about their ethnicity, migration status, job role, and whether or not they were redeployed. Outcome 1, a binary variable, is whether or not a HCW was redeployed to a different role because of the pandemic. Outcome 2, a binary variable is whether or not a HCW received training during redeployment. Outcome 3, a binary variable, is whether or not a HCW received supervision during redeployment. Outcome 4, a binary variable is whether direct patient contact decreased, remained the same or increased in HCW's redeployed role. Outcome 5, a binary variable, is whether or not a HCW interacted with patients with COVID-19. Derivation of all outcomes is described in detail in Figure 1. In the occupation variable, nursing includes midwives and nursing associates; HCW, healthcare workers; NHS, National Health Service.

#### Interaction with COVID-19 patients among redeployed HCWs

Those from Black (1.33, 1.07–1.66, *p* = 0.009) and Asian (1.30, 1.14–1.48, *p* < 0.001) ethnic backgrounds were significantly more likely to report interaction with COVID-19 patients than White HCWs (Figure 4 and Supplementary Table S4). Similarly, HCWs born outside the UK (1.27, 1.13–1.42, *p* < 0.001), as opposed to those born in the UK were more likely to report increased interaction with COVID-19 patients.

### Sub-cohort analyses

Supplementary Table S3 describes the cohort based on their level of seniority using the AfC pay band and doctors’ grades as proxy measures for occupational seniority. About 19% of HCWs on the AfC pay scale worked in Band 6 (*n* = 2029), and 9% of doctors held consultant positions (*n* = 966).

#### Redeployment

Among those on the AfC pay scale, we found that HCWs in senior positions, like Band 8 or 9 (0.69, 0.55–0.87, *p* = 0.001), in comparison to junior roles like Band 5, were less likely to report redeployment. Further, HCWs born in the UK were less likely to report redeployment than those born abroad (1.26, 1.06–1.49, *p* = 0.01) (Supplementary Table S6 and Supplementary Figure S2).

After adjusting for doctor's grade, consultants were significantly less likely to report being redeployed in comparison to foundation-year doctors (3.54, 2.15–5.83, *p* < 0.001). Additionally, doctors from a Black ethnic background were less likely to report redeployment (0.58, 0.37–0.92, *p* = 0.02) compared to their White colleagues (Supplementary Table S5 and Supplementary Figure S1).

#### Training and supervision

Doctors of Asian background (0.55, 0.34–0.87, *p* = 0.01) were less likely to report receiving training for their redeployed role, however, this effect was not observed in Asian HCWs within the AfC pay scale. Doctors from a Black background (5.41, 1.10–26.60, *p* = 0.04) were significantly more likely to report receiving supervision compared to their White colleagues, which again was not evident within HCWs on the AfC pay scale (Supplementary Table S5 and Supplementary Figure S3).

#### COVID-19 contact

After adjusting for grade, the significant effect of ethnicity on COVID-19 patient contact diminished, however, doctors born overseas were more likely to report contact with COVID-19 patients (1.40, 1.14–1.72, *p* = 0.001) (Supplementary Table S5 and Supplementary Figure S4). Among HCWs on the AfC pay scale, Black (1.62, 1.15–2.27, *p* = 0.006) and Asian (1.24, 1.00–1.52, *p* = 0.05) HCWs were significantly more likely to report COVID-19 contact. (Supplementary Table S6 and Supplementary Figure S5).

## Discussion

### Principal findings

Our study, the largest of its kind, analyses data from a diverse sample of over 10,000 HCWs in the UK and sheds light on variations in redeployment experiences during COVID-19. In our knowledge, this is the first analysis to show migrant HCWs on AfC pay bands were more likely to be redeployed than their UK-born counterparts. Furthermore, ethnic minority HCWs, especially those from Asian and Black backgrounds, faced disparities in training, supervision, and patient contact during redeployment, with Asian HCWs reporting lesser training and Black HCWs more supervision compared to their White counterparts. These findings underscore the disproportionate challenges faced by ethnic and migrant HCWs during redeployment.

### Strengths and limitations

The strengths of this study include its large and diverse sample, along with extensive phenotyping of our cohort, allowing for a detailed examination of redeployment experiences across different demographic and occupational groups. However, the cross-sectional design limits us from determining causality, and there is potential of recall bias, as participants may have retrospectively exaggerated poor experiences. Moreover, the broad definition of migrants used in this study, while necessary to maintain statistical power, may obscure differences between recent and long-term migrants. Volunteer bias is also a consideration as with any consented cohort study, though the sample is largely representative of the NHS workforce, albeit with a lower proportion of ancillary staff.^28^

Lastly, our findings relating to COVID-19 contact during redeployment should be interpreted with caution. While the questionnaire items relating to HCWs' redeployment and their interaction with COVID-19 patients inquire about similar periods (‘during the UK national lockdown’ for redeployment and ‘in the first month after the start of UK national lockdown’ for COVID-19 interaction), it is possible that participants were redeployed after the first month and thus could have been reporting COVID-19 contact in their usual role. Given that most of the redeployment in the NHS occurred in March and April 2020, we assume that reports of COVID-19 contact relate to the redeployed role.^29,30^

### Relation to other studies

Our findings align with existing literature,^18,31^ which suggests that those in nursing roles had higher likelihood of being redeployed compared to those in medical roles, likely due to differences in skill sets and the nature of patient care responsibilities. The increased patient contact among redeployed HCWs in nursing roles has been previously linked to higher occupational exposure to SARS-CoV-2 and greater risk of infection, adversely impacting their physical and mental well-being due to elevated stress and anxiety levels.^32,33^ Further, our study adds to the growing body of evidence highlighting systemic bias within the NHS, particularly affecting migrant HCWs, who were more likely to be redeployed than their UK-born counterparts.^31^ Additionally, HCWs from ethnic minority backgrounds reported inadequate training and were more likely to report interaction with COVID-19 patients, despite limited changes to their working practices post-risk assessments.^34^ This could be linked to structural discrimination, as existing literature extensively documents that HCWs from ethnic minority backgrounds often felt less empowered to voice concerns about risk assessments, and had less autonomy in decisions related to their redeployment.^31,35^ Black HCWs, especially doctors, were significantly more likely to report receiving supervision compared to their White counterparts. While clinical supervision has various benefits, including reduced stress and anxiety, better quality of care delivery, and better working environment,^36^ it is essential to address any negative connotations associated with supervision among ethnic minority staff, such as being supervised due to a lack of trust in their experience and skills, or as a form of micromanagement. Persistent structural discrimination within the NHS has been well-documented.^9,31,37^ The disparities in redeployment, COVID-19 contact, training and supervision based on ethnicity, migration and occupational role, as highlighted in our study, warrant further investigation in the context of structural discrimination. Using an intersectional lens will enable a better understanding of how these disparities proliferate.

### Meaning of the study

The implications of this study are significant for workforce management, particularly during emergencies like the COVID-19 pandemic. It is crucial for policymakers to consider the specific needs and risks of individual HCWs before redeploying them to high-risk roles. While redeployment is an important strategy for workforce management during emergencies, we believe it is essential to consider individual HCWs’ training and supervision needs, as well as their risk profiles, before redeploying them to roles that may increase their exposure to high-risk conditions. Even post-pandemic, the NHS is experiencing a severe backlog of care, exacerbated by staffing shortages.^38,39^ As HCWs continue to be reassigned or redeployed to different wards, our findings are relevant not only for future emergency responses, but also for routine workforce management. The inequalities in redeployment experiences could have potentially influenced HCWs’ intentions to leave the NHS workforce, making our findings crucial for informing workforce policy development at a time of significant staffing shortages. We also believe it is crucial to address the impact of racism and structural discrimination within the NHS, which could result in the disproportionate redeployment of certain groups HCWs, as also pointed by previous research.^40^ This will ultimately foster greater inclusivity of ethnic minority staff and protect them in times of crises, creating a more supportive environment and ultimately improving patient care.

### Unanswered questions and future research

While our study captures differences in redeployment experiences, qualitative data is required to capture the complexity of redeployment experiences to better understand the context and conditions under which redeployment took place and HCWs agency to accept or refuse redeployment decisions and the transparency of decision-making. More evidence is also needed to explore the link between redeployment during the pandemic and staff attrition, which could provide valuable insights for staff retention policies.

## Supplemental Material

sj-docx-1-shr-10.1177_20542704241290721 - Supplemental material for Redeployment experiences of healthcare workers in the UK during COVID-19: a cross-sectional analysis from the nationwide UK-REACH study^*^ Supplemental material, sj-docx-1-shr-10.1177_20542704241290721 for Redeployment experiences of healthcare workers in the UK during COVID-19: a cross-sectional analysis from the nationwide UK-REACH study^*^ by Zainab Zuzer Lal, Christopher A. Martin, Mayuri Gogoi, Irtiza Qureshi, Luke Bryant, Padmasayee Papineni, Susie Lagrata, Laura B Nellums, Amani Al-Oraibi, Jonathon Chaloner, Katherine Woolf and Manish Pareek in JRSM Open

sj-docx-2-shr-10.1177_20542704241290721 - Supplemental material for Redeployment experiences of healthcare workers in the UK during COVID-19: a cross-sectional analysis from the nationwide UK-REACH study^*^ Supplemental material, sj-docx-2-shr-10.1177_20542704241290721 for Redeployment experiences of healthcare workers in the UK during COVID-19: a cross-sectional analysis from the nationwide UK-REACH study^*^ by Zainab Zuzer Lal, Christopher A. Martin, Mayuri Gogoi, Irtiza Qureshi, Luke Bryant, Padmasayee Papineni, Susie Lagrata, Laura B Nellums, Amani Al-Oraibi, Jonathon Chaloner, Katherine Woolf and Manish Pareek in JRSM Open
